# “The Good into the Pot, the Bad into the Crop!”—A New Technology to Free Stem Cells from Feeder Cells

**DOI:** 10.1371/journal.pone.0003788

**Published:** 2008-11-21

**Authors:** Annette Schneider, Dimitry Spitkovsky, Peter Riess, Marek Molcanyi, Naidu Kamisetti, Marc Maegele, Jürgen Hescheler, Ute Schaefer

**Affiliations:** 1 Institute for Research in Operative Medicine (IFOM), Faculty of Medicine, University of Witten/Herdecke, Witten, Germany; 2 Center of Physiology, Institute of Neurophysiology, Medical Faculty, University of Cologne, Cologne, Germany; 3 Department of Trauma and Orthopedic Surgery, Faculty of Medicine, University of Witten-Herdecke at the Hospital Merheim, Cologne, Germany; 4 Clinic of Neurosurgery and 2nd Department of Neurophysiology, Faculty of Medicine, University of Cologne, Cologne, Germany; 5 Department of Experimental Neurotraumatology, Medical University Graz, Graz, Austria; Columbia University, United States of America

## Abstract

A variety of embryonic and adult stem cell lines require an intial co-culturing with feeder cells for non-differentiated growth, self renewal and maintenance of pluripotency. However for many downstream ES cell applications the feeder cells have to be considered contaminations that might interfere not just with the analysis of experimental data but also with clinical application and tissue engineering approaches. Here we introduce a novel technique that allows for the selection of pure feeder-freed stem cells, following stem cell proliferation on feeder cell layers. Complete and reproducible separation of feeder and embryonic stem cells was accomplished by adaptation of an automated cell selection system that resulted in the aspiration of distinct cell colonies or fraction of colonies according to predefined physical parameters. Analyzing neuronal differentiation we demonstrated feeder-freed stem cells to exhibit differentiation potentials comparable to embryonic stem cells differentiated under standard conditions. However, embryoid body growth as well as differentiation of stem cells into cardiomyocytes was significantly enhanced in feeder-freed cells, indicating a feeder cell dependent modulation of lineage differentiation during early embryoid body development. These findings underline the necessity to separate stem and feeder cells before the initiation of *in vitro* differentiation. The complete separation of stem and feeder cells by this new technology results in pure stem cell populations for translational approaches. Furthermore, a more detailed analysis of the effect of feeder cells on stem cell differentiation is now possible, that might facilitate the identification and development of new optimized human or genetically modified feeder cell lines.

## Introduction

Optimal standardized culture conditions for adult and embryonic stem cells that maintain multipotency or pluripotency, self-renewal and transplantability are paramount for high quality translational research and clinical application. Several culturing parameters that maintain these main characteristics of stem cells may also be detrimental. For instance, co-culturing of stem cells with animal derived feeder cells has been shown to present the risk of contamination with retroviruses or other pathogens that could be transmitted to patients and the wider population [1], [2].

To minimize the risk of transmitting infectious diseases from animals strategies have been developed that omit nonhuman factors from stem cell cultures, i.e. a variety of human feeder cell cultures were identified and analysed for the maintenance of human stem cells [3], [4], [5].

But even the use of human feeder cell cultures for the maintenance of human stem cells might prove a double edged sword, since feeder layers may also limit the interpretation of research results. Experimental data may result from combined stem cell and feeder cell responses [6]. Our own data demonstrated that the increased level of neurotrophic factors in medium following conditioning of murine embryonic stem cells with rat brain extract was due in equal shares to the release of these factors from stem cells as well as from feeder cells [7].

Consequently, feeder free culture systems were developed for the maintenance and expansion of stem cells [8]. In feeder free culture systems the complex environment provided by feeder cells is either substituted by a range of extrinsic factors [9], [10], [11], [12] or by addition of feeder-cell conditioned medium. Proteomic analysis of feeder-cell conditioned medium highlighted the complexity of the stem cell niche provided by feeder cells. 136 unique protein species were identified in medium from mouse embryonic fibroblast feeder cells which support the growth of human embryonic stem cells [13].

To account for the complexity of the feeder cell associated environment and allow for the separate analysis of stem and feeder cells, stem cells were co-cultured with microencapsulated feeder cells in a novel approach [14].

However, the impact of direct interaction between feeder and stem cells on stem cell expansion and differentiation is not addressed by this approach. The effect of cell-cell contact on stem cell maintenance and multipotentiality has predominantely been demonstrated for haematopoietic progenitor cells. Direct contact between hematopoietic progenitor cells (HPC) and the cellular microenvironment seem to be essential for maintaining “stemness”. Furthermore, direct interaction between HPC and multipotent mesenchymal stromal cells appears beneficial for the expansion of HPCs harboring CD133(+) [15]. Similar beneficial effects were observed in adult stem cells supported by feeder cell. When neural stem cells were cultured on stem cell monolayers, their proliferation was decreased, but neuronal differentiation was significantly induced. Direct physical contact between human adipose tissue stromal cells (hATSCs) and neural stem cells (NSCs) was required for the induction of neuronal differentiation [16].

Moreover, cellular interaction via gap junctions is thought to play an important role in embryonic cell survival and differentiation [17]. Less pronounced intercellular communication was also observed between human embryonic stem cells and mouse embryonic feeder cells [17].

These studies indicate that cell-cell contact between feeder cells and stem cells as well as soluble factors released by feeder cells may contribute to stem cell maintenance and growth.

The aim of the present study was to establish a method that provides optimal conditions for stem cell cultivation, such as the co-cultivation of feeder and stem cells during the proliferative phase, and at the same time allows for the analysis and transplantation of feeder free stem cells.

## Methods

### Cell culture conditions

MEFs (Neomycin-Resistant Mouse PEF Day 13.5 post coitum, (Cellsystems, St. Katharinen, Germany)) were mitotically inactivated with Mitomycin (Serva, Heidelberg, Germany) 10 mg/ml, 2 hours.

Murine embryonic stem cells (D3) or D3 derived cells engineered to express EGFP under control of the α-myosin heavy chain promoter (were expanded on MEF feeders (7.5×10^4^ cells per cm^2^). Cultures were maintained in DMEM supplemented with 15% FCS, 2 mM L-Alanyl-L-Glutamine (Biochrom AG, Berlin, Germany), 1% NEAA (Biochrom), 100 µM β-ME (Serva), and 10 ng/ml human recombinant leukemia inhibitory factor (Chemicon, Temecula, CA).

### Automated aspiration of stem cell colony fractions from feeder cell layer

Stem cell colony fractions were aspirated with the CellCelector™ Robot System (Aviso, Greiz, Germany). By combining imaging of clones/cells with a harvesting process that is based on predefined parameters such as cell size, morphology, spectral features and/or intensitiems, the automated robotic system used enables the selection and aspiration of specific cell colonies or part of cell colonies. Scanning and definition of specific colonies/cells is supported by an inverted microscope (Olympus).

Colonies of stem cell line D3 were grown to an approximate size of 500 µm in diameter on mitomycin treated MEF and colony fractions were aspirated with a glass capillary tool (aperture: ∅ 80 µm). For automated selection following parameter thresholds of colonies were defined: area: ∅>400 µm; sphericity 1–0.8; convexity: 0.8–1; elongation: 1–5; shape factor: 0.8. Standard aspiration conditions included: elevation of glass capillary (single cell module) aperture: 50 µm above tissue culture plate surface; aspiration volume: 1 µl; aspiration speed: 7%; waiting time: 1 sec. Each aspirated cell colony fraction was dispensed (dispension speed: 25%) into a corresponding well of a V-96 well plate. Each well contained 20 µl proliferation media (DMEM supplemented with 15% FCS, 2 mM L-Alanyl-L-Glutamine, 1% NEAA and 100 µM β-ME). Aspiration tool was automatically rinsed once after every dispension. Automated documentation of data with the Soft Imaging System Cell^D^ included particle maps, particle results, overview/scans, particle before pick and particle after pick. Automated microscopic imaging was performed with the integrated Olympus CXK 41 microscope.

### Embryoid Body Formation

Embryoid body formation was initiated either from stem cells grown on mitomycin treated feeder cells or stem cell colony fractions aspirated from feeder cell monolayer. For initiation of embryoid body formation in hanging drops cells were dissociated by 0.05% trypsin and 0.04% EDTA in PBS and collected by centrifugation. Subsequently the numbers of viable cells were determined. 200 cells/20 µl medium (DMEM supplemented with 15% FCS, 2 mM L-Alanyl-L-Glutamine, 1% NEAA and 100 µM β-ME) were transferred onto a lid of a Petri dish and incubated upside down or into individual wells of a V-96 well plate. For embryoid body formation from feeder-freed stem cells each feeder-freed stem cell colony fraction was transferred to a single well of untreated V-96 plates and cultured for 4 days in 100 µl of medium. After 4 days 100 µl additional medium was added to ensure adequate nutritional supply for the growing embryoid bodies. For analysis of differentiation into the cardiomyocyte lineage 10 ng/ml human recombinant leukemia inhibitory factor was added to the medium during embryoid body formation.

### Growth curves

The size of individual embryoid bodies seeded in hanging drops or V-96 wells were determined at different time points using Leica application suite Software of Leica DMI400B microscope.

### Neuronal differentiation

#### Selection of nestin-positive cells

Embryoid bodies kept in suspension culture for 4 days were plated onto a tissue culture surface coated with Gelatin. A 24 h incubation in DMEM (Biochrom AG)-10% FCS (Sigma-Aldrich, Munich, Germany) medium allowed EBs to spread on this substrate. The next day the medium was switched to DMEM/F12 (1∶2) (Biochrom AG) supplemented with insulin (5 µg/ml), transferrin (50 µg/ml), selenium chloride (30 nM), and fibronectin (5 µg/ml) (all (Sigma-Aldrich) (ITSFn medium). The culture medium was replenished every second days. A maximal number of nestin-positive cells appeared approximately 6–8 days after replacement with ITSFn medium.

#### Expansion of nestin-positive cells by bFGF

Cells maintained in ITSFn medium were dissociated by 0.05% trypsin and 0.04% EDTA in PBS (Biochrom AG), neutralized with DMEM/F12 (1∶2) plus 10% FCS (Sigma-Aldrich), collected by centrifugation, and replated at a cell density of 0.5–2×10^5^/cm^2^ on dishes precoated with polyornithine (15 mg/ml) and laminin (l µg/ml) (both Sigma-Aldrich). Culture medium was DMEM/F12 supplemented with insulin (25 µg/ml), transferrin (50 µg/ml), progesterone (20 nM), putrescine (100 µM), selenium chloride (30 nM), bFGF (5 ng/ml)(Sigma-Aldrich), and laminin (1 µg/ml) (N3 medium). The medium was changed every 2 days. For passage, cells were dissociated by 0.05% trypsin and 0.04% EDTA in PBS, collected by centrifugation, and replated.

#### Differentiation of nestin positive cells into the neuronal lineage

Proliferating nestin-positive cells were switched to N3 medium without bFGF and cultivated for 6–15 days. Differentiation of nestin positive cells into neuronal cell types was evaluated by expression analysis of specific neuronal markers.

### RT-PCR Analysis

Total RNA was prepared from trypsinized cells using High Pure RNA isolation kit (Roche Diagnostics GmbH, Mannheim, Germany) according to the manufacturer's instructions. Reverse transcription and polymerase chain reaction amplification was performed in an Eppendorf Mastercyclergradient (Eppendorf, Hamburg, Germany). Reverse transcription was prepared with RevertAid First Strand cDNA Synthesis Kit (Fermentas Inc. Burlington, Canada) and done in a volume of 20 µl using 1 µg of total RNA according to the manufacturer's instructions. 25 µl PCR reaction contained 0.2 µl cDNA, 1 U (PeqGold Taq-DNA Polymerase (Peqlab, Erlangen, Germany) 2.5 mM dNTP mix, 50 mM MgCl_2_ and 10 pM/µl of each sense and anti-sense primer. To determine mRNA expression of differentiation markers, amplification was performed in 35–40 cycles (30 sec at 94°C, 30 sec at the appropriate annealing temperature, 1 min at 72°C). To perform a semiquantitative analysis, different cycle numbers were used for the respective primer set in order to remain below the plateau phase of amplification.

For nested PCR 1 µl DNA amplificated from neomycin resist. primers were transfered into a 25 µl PCR reaction containing 0.2 µl cDNA, 1 U (PeqGold Taq-DNA Polymerase (Peqlab, Erlangen, Germany) 2.5 mM dNTP mix, 50 mM MgCl_2_ and 10 pM/µl of each sense and anti-sense nested neomycin resist. primer. Amplification was performed at 57°C in 40 cycles.

Sense and anti-sense oligonucleotide primers were synthesized according to the gene sequences extracted from a gene bank (Table 1).

**Table 1:** Primer sequences and optimized annealing temperatures (AT).

Gene name		AT °C	bp	sense primer	antisense primer
neomycin resist. (NR)	FJ040214	57	415	CGACAAGACCGGCTTCCATC	TGGGTGGAGAGGCTATTCGG
NR (nested RT-PCR)	FJ040214	57	323	TGGGTGGAGAGGCTATTCGG	CGACAAGACCGGCTTCCATC
oct-4	X52437	54	593	AGAGGGAACCTCCTCTGAGC	CTG GGA AAG GTG TCC CTG TA
fgf5	NM_010203	58	456	CTTCAGTCTGTACTTCACTGG	AAAGTCAATGGCTCCCACGAA
gata6	NM_10258	58	574	CTCTGGGTAGCACCAGCTCA	GCAATGCATGCGGTCTCTAC
T brachyury	NM_009309	58	947	CCAGGTGCTATATATTGCCT	TGCTGCCTGTGAGTCATAAC
gata4	NM_008092	60	107	TCAAACCAGAAAACGGAAGC	GTGGCATTGCTGGAGTTACC
cTnC	NM_009393	60	124	CAGCAAAGGGAAGTCTGAGG	CGTAATGGTCTCACCTGTGG
alpha-MHC	NM_010856	60	120	GATGGCACAGAAGATGCTGA	CTGCCCCTTGGTGACATACT
nestin	NM_016701	58	434	CTCGGGAGAGTCGCTTAGAG	ATT AGG CAA GGG GGA AGA GA
neurofilament	NM_010910	54	625	TGAGCTGAGAAGCACGAAGA	TTG GTT GGT GAT GAG GTT GA
NSE	NM_013509	56	301	CTTCCTTCACCAGCTCCAAG	CTCTATCGCCACATTGCTCA
GAPDH	NM_199472.1	58	183	AGAACATCATCCCTGCATCC	CCT GCT TCA CCA CCT TCT TG

### Immunocytochemistry

Following differentiation of embryonic stem cells into cells of neuronal cell lineage, cells were fixed for 5 min in 4% paraformaldehyde/0.15% picric acid and permeabilized for 15 min in 0.2% Triton X-100. Subsequently, cells were incubated with primary antibodies diluted in PBS for 16 h at 4°C. Slides were rinsed in PBS and then incubated with secondary antibodies diluted in PBS for 1 h at room temperature.

Primary antibodies used were: mouse anti-NeuN (1∶15, Chemicon, Temecula, CA), anti-mouse nestin (1∶100, Acris, Hiddenhausen, Germany), anti-mouse NSE (1∶50, Acris), mouse anti-Map2 (1∶1000, Abcam, Cambridge, UK)

Secondary antibodies: anti-mouse IgG Alexa 350 conjugated (1∶100, Invitrogen, Oregon UK) anti-chicken IgG Alexa 555 conjugated (1∶300, Invitrogen) and anti-rabbit IgG Chromeo 488 conjugated (1∶1250, Abcam).

The labeled cells were subsequently viewed with a fluorescent (Leica DMI400B) microscope (Leica Microsystems, Heerbrugg, Switzerland) at 350–555 nm; images were captured using the Leica application suite Software.

### Data Analysis

For statistical analysis of RT-PCR results obtained with cellular systems a minimum of three experiments were performed. The expected variance of RT-PCR results should allow the detection of statistically significant differences. RT-PCR results were normalized against internal controls (GAPDH) before statistical analysis was performed. Differences in values of two groups were tested using the paired Student́s t-test. When appropriate Levene's test for equality of variances was performed and subsequently accounted for in the statistical analysis. For multiple comparisons statistical significance was evaluated using ANOVA analysis. Tests were two-sided and only p-values ≤0.01 were considered significant. The results are displayed as means (±S.E.).

## Results

### Feeder-cell contamination during neuronal differentiation of embryonic stem cells

**Figure 1:**
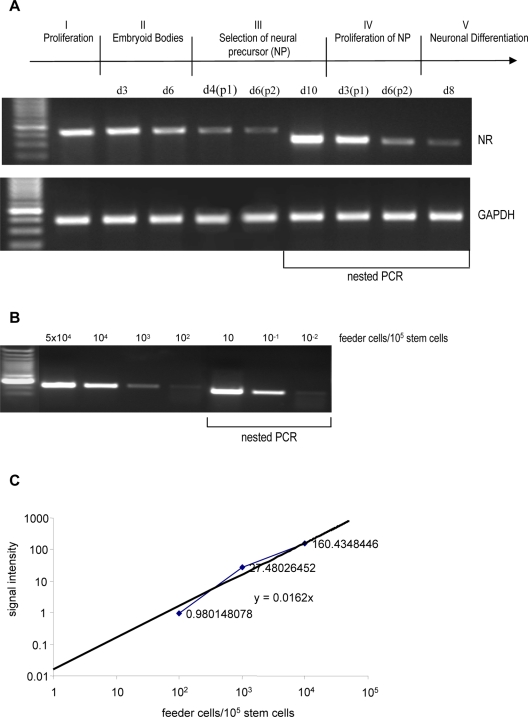
Analysis of feeder cell contamination during neuronal differentiation of murine embryonic stem cells. Feeder cells were transfected with the neomycine resistance (*nr*) gene. A. RT-CR and nested RT-PCR analysis of *nr* gene expression (NR) during neuronal differentiation of murine ESCs. Displayed are images of representative results following RT-PCR analysis. GAPDH was used as a control. B. Percentage of feeder cell contamination was evaluated by *nr* gene expression analysis of predefined ratios of stem and feeder cells. Displayed are representative results of PCR products. C. Following logarithmic transformation signal density is plotted as a linear function against feeder cell number (n = 4).

Murine embryonic stem cells (D3) were cultured on a layer of mitomycin treated mouse embryonic fibroblast feeder cells. We have used mouse embryonic fibroblast feeder cells transfected with the neomycin resistance gene in order to detect feeder cell contaminations. Embryonic stem cells were differentiated into neuronal cells by standard procedures. Cells were harvested from stage I–V as indicated (I/Proliferative Phase; II/Embryoid Bodies; III/Selection of Neural Precursors; IV/Proliferation of Neural Precursors; V/Neuronal Differentiation). Expression of the neomycin resistance (*nr*) gene was quantified by RT-PCR or nested RT-PCR analysis. Cells were passaged twice in phase III and IV. Embryonic stem cells differentiated into neuronal cells by standard procedures were contaminated by feeder cells up to phase V as detected by *nr* gene expression (Figure 1A). RT-PCR analysis of *nr* gene contamination in phase IV and V was detected by nested RT-PCR. In order to estimate the sensitivity of the RT-PCR and nested RT-PCR analysis and to quantify the percentage of contamination *nr* expression was analysed for predefined ratios of stem and feeder cells. Following logarithmic transformation signal density of RT-PCR results was plotted as a linear function against feeder cell number. Signal intensity of 10^2^–10^4^ feeder cells/10^5^ stem cells were shown to be in the linear range and therefore used to calculate the regression line. A regression equation was calculated (y = 0.0162x) to predict feeder cell numbers from signal intensities of unknown feeder/stem cell ratios during neuronal differentiation. The nested RT-PCR analysis allowed the detection of 0.001% contamination of feeder cells (1 feeder cell /10^5^ stem cells) (Figure 1B). In embryoid bodies up to 7% (day3: ∼7×10^3^ feeder cells/10^5^ stem cells; day 6: ∼6×10^3^/10^5^ stem cells) feeder cell contamination was demonstrated (Figure 1C). In the early stages of neural precursor cell selection (phase III) an average of 2% mouse embryonic fibroblast contamination (2×10^3^ feeder cells/10^5^ stem cells) was still detected, indicating a level of contamination that could impair the interpretation of research results or impede the successful implementation of precursor based regenerative therapies. The calculated percentages exemplify the potential of feeder cell contaminations during stem cell cultivation and differentiation and might vary considerably dependent on seeding conditions.

**Figure 2:**
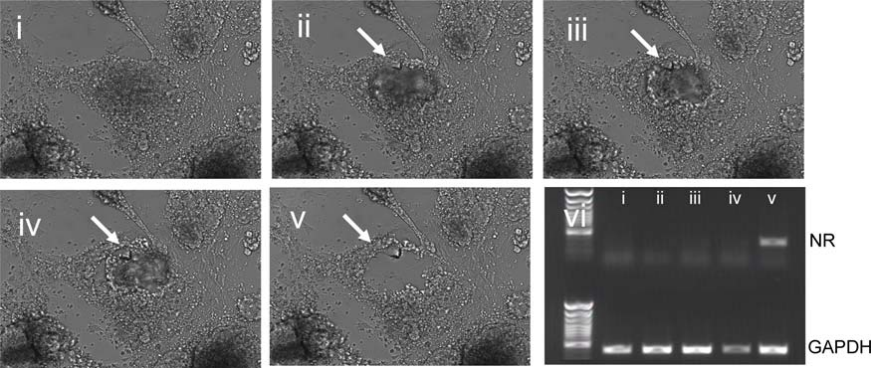
Automated aspiration of stem cell colony fractions from feeder cell monolayers. Panel i.–v.: Automated documentation of a representative aspiration procedure using the CellCelector™/Soft Imaging System Cell^D^. lane i.–v.: Determination of feeder cell contamination by RT-PCR expression analysis of nr mRNA.

Stem and feeder cells were separated by automated aspiration of stem cells with a single cell glass capillary module. The volume of aspirated stem cell suspension was determined by defined aspiration pressure and module height. At a volume of 1 µl cell suspension, an aspiration speed of 7% and 50 µm elevation of the aspiration aperture extraction of cells resulted in the complete removal of feeder cells as determined by neomycin resistance gene expression (Figure 2, panel i, lane i). Aspiration of stem cells was repeated at the same colony site with the same preset aspiration pressure and module height up to five times before feeder cell contamination was detected (Figure 2, panel ii–v, lane ii–v). The number of repeated cell aspirations from one colony resulting in pure feeder-freed stem cell (*f-f*SC) might vary and depends on colony size. However following a singular aspiration step with the above described preset conditions stem cell contamination with feeder cells was never observed.

### Embryoid body formation from feeder-freed stem cell colony fractions

**Figure 3:**
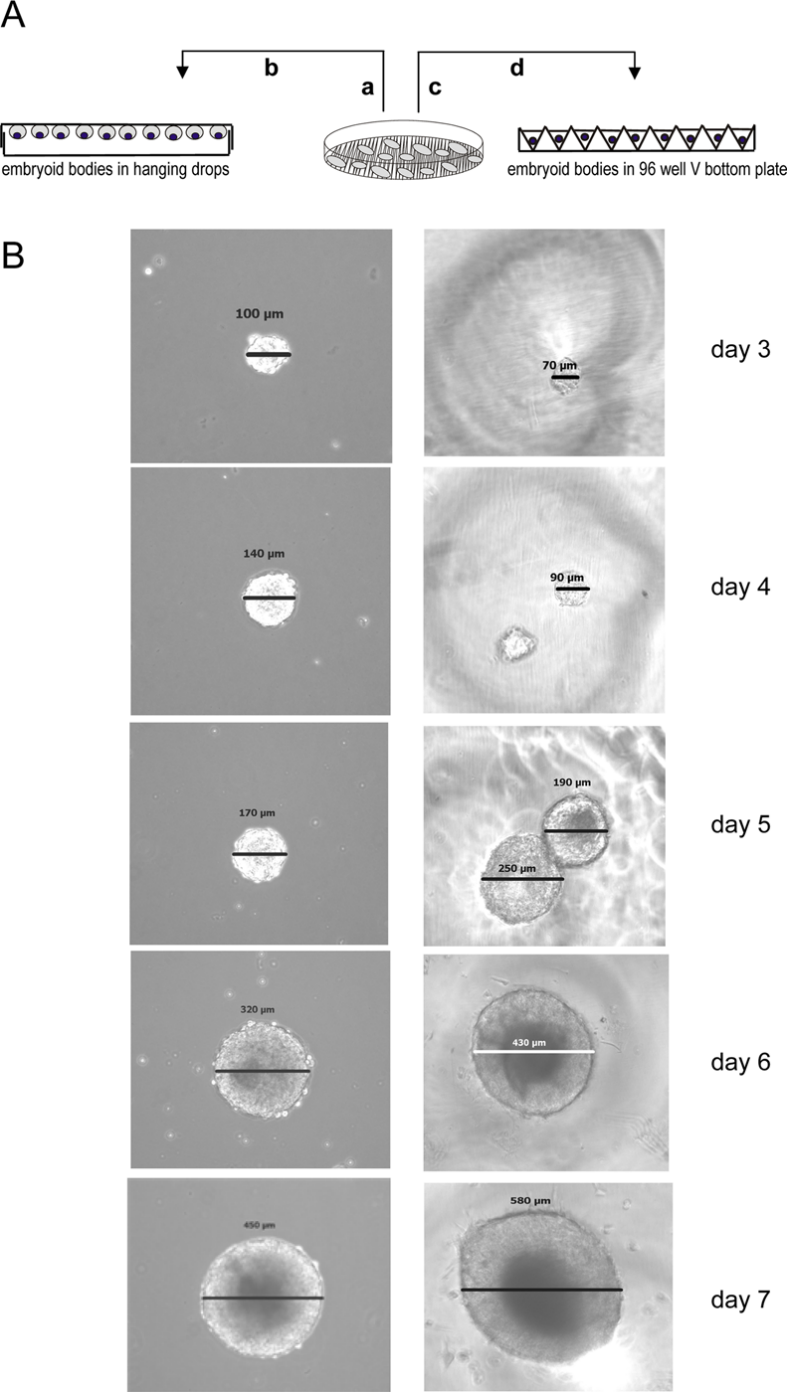
Embryoid body formation using standard conditions or feeder–freed stem cells. A. Schematic representation of embryoid body formation: a. trypsination of stem/feeder cells, b. standard protocol for embryoid body formation in hanging drops, c. automated harvesting of feeder-freed stem cells colony fractions, d. transfer of feeder-freed stem cell to V-96 plate. B. Microscopic images of time dependent growth of (feeder-freed) *f-f* EBs and (standard) *st*EBs.

**Figure 4:**
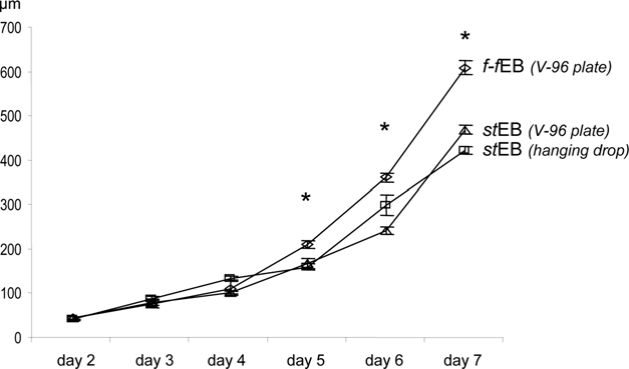
Quantitative analysis of time dependent increase in cross sectional dimensions of *f-f*EBs and *st*EBs (±SEM; n = 20). *diamant*: *f-f*EBs; *triangle*: *st*EBs in hanging drops; *square*: *st*EBs in V-96 wells.

Formation of embryoid bodies from feeder-freed colony fractions (*f-f*EBs) was compared to EBs generated by commonly used culture protocols (*st*EB). In order to examine the development of embryoid bodies from distinct feeder-freed stem cell colony fractions each feeder-freed stem cell colony fraction was transferred to a single well of untreated V-96 plates, (Figure 3A). Within the first 3 days cross sectional dimensions of single *f-f*EBs in V-96 wells were smaller or comparable to *st*EBs grown in hanging drops (Figure 3B). After day 5 the cross sectional dimensions of *f-f*EBs increased significantly as compared to *st*EBs contaminated with feeder-cells. By day 7 *f-f*EBs were 30% larger than *st*EBs in suspension culture and 23% larger than *st*EBs in V-96 plates (day 7; *f-f*EBs: 618.5±17 µm; *st*EBs: 431.1±9 µm, *st*EBs (V-96): 478.5±10; *p*≤0.01), indicating an inhibitory effect of feeder-cells on embryoid body growth (Figure 4). It has to be noted that the range of sizes observed in *f-f*EBs at different time points differed significantly as compared to the range of size of *st*EBs (day 7: *f-f*EBs: 450–700 µm; *st*EBs: 380–470 µm; Levene's test for equality of variances *p*<0.015), indicating that a varying number of cells were aspirated during the automated separation process. The diversity in range size of *f-f*EBs was accounted for in the statistical analysis of results. It has been shown for human stem cells that cross sectional dimensions of EBs and/or the number of primarily seeded stem cells might affect lineage determination [18] and differentiation efficacy [19], we have therefore subsequently analysed the pluripotent differentiation potential of *f-f*EBs.

### Neuronal and cardiomyocyte differentiation potential is unaffected following separation of stem and feeder cells

**Figure 5:**
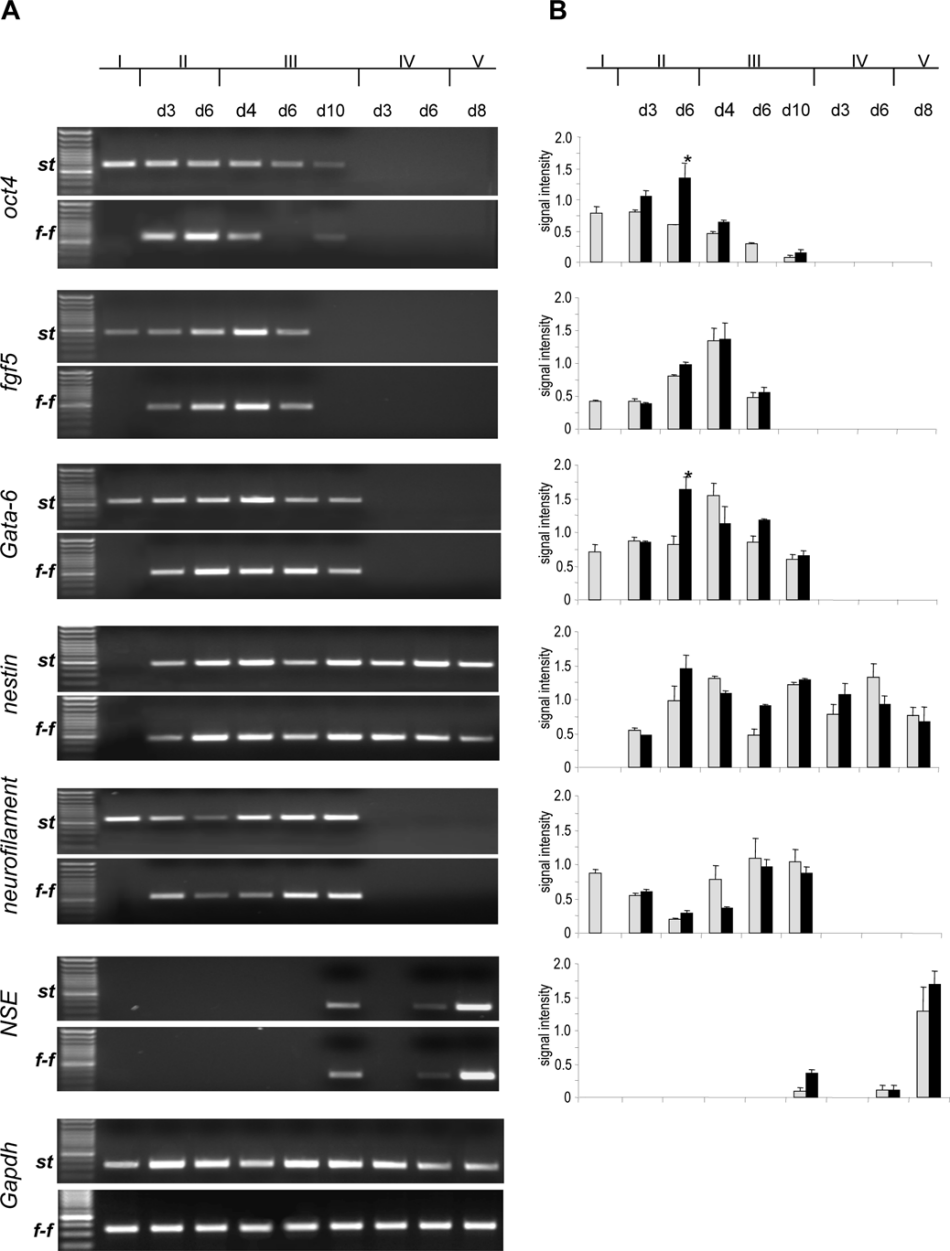
Gene expression analysis of neuronal differentiation markers by semi-quantitative RT-PCR. A. Displayed are images of representative results following RT-PCR analysis. B. Quantitative analysis of neuronal and cell lineage marker expression in feeder-freed stem cells (*f-f*) and stem cells grown under standard conditions (*st*) (±SEM, n = 6). *grey: st*SCs; black: *f-f*SCs.

**Figure 6:**
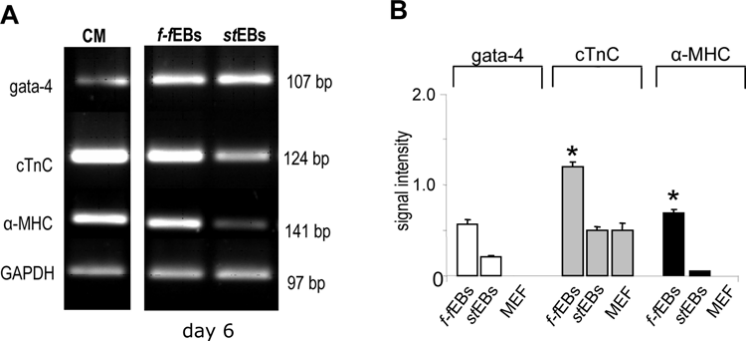
Gene expression analysis of cardiomyocyte differentiation markers by semi-quantitative RT-PCR. A. Images of representative results of PCR products following amplification of cardiomyocyte differentiation markers. B. Quantitative analysis of gene expression of cardiomyocyte differentiation markers in *f-f*EBs and *st*EBs at day 6 (±SEM, n = 3). *white*: gata-4, *grey*: C TnC (*cardiac Troponin C*) *black*: αMHC (*α-myosin heavy chain*), CM: cardiomyocyte; MEF: murine embryonic fibroblasts (feeder cells).

At day 4 after stem cell seeding *f-f*EBs and *st*EBs were differentiated into neurons by standard protocols. Time dependent neural/neuronal differentiation of embryoid bodies was evaluated by RNA expression analysis of stem cell marker *oct4*, marker of neuroectodermal (*fgf5*), endodermal (*gata-6*) and mesodermal (*brachyury*) lineage as well as neural (*nestin*) and neuronal (*NSE*, neurofilament) markers. As a control marker expression was also evaluated in feeder cells alone (Figure 5A, B). In feeder cells marginal expression of only *oct4* (0.5±0.2; signal intensity; SEM), *fgf* (0.7±0.1; signal intensity; SEM), *gata-6* (0.4±0.06; signal intensity; SEM) and *nestin* (0.3±0.2; signal intensity; SEM) was detected (results not shown). In *f-f*EBs expression of the stem cell marker o*ct4* was significantly elevated after 6 days of culturing in V-96 plates as compared to *st*EBs in suspension culture. These findings are in accordance with the significant proliferation of *f-f*EBs observed at this time point (see above). Early expression of lineage markers was demonstrated for *fgf5* and *gata-6* but not for *brachyury.* G*ata-6* an endodermal differentiation marker that has also been shown to be expressed in cardiac mesoderm [20] is significantly elevated in *f-f*EBs during late EB formation (day 6). *Gata-6* expression ceased at day 10 (stage III, selection of neural precursors). In order to ascertain the implication of these observations we have analysed the expression of cardiomyocyte markers such as *gata-4*, *cardiac Troponin C* (*cTnC*) and *α-myosin heavy chain* (*α-MHC*) in *f-f*EBs and *st*EBs at the corresponding stage of embryoid body formation (Figure 6A, B). Early cardiomyocyte marker *gata-4*, *cTnC* and *α-MHC* expression was significantly higher in *f-f*EBs than in *st*EBs (*gata-4*: p>0.002; *cTnC*: p<0.001; *α-MHC* p<0.001) (Figure 6B). *cTnC* and *α-MHC* expression in *f-f*EBs was comparable to expression of these markers in cardiomyocytes, indicating an inhibitory effect of feeder cells on cardiomyocyte differentiation during EB formation.

**Figure 7:**
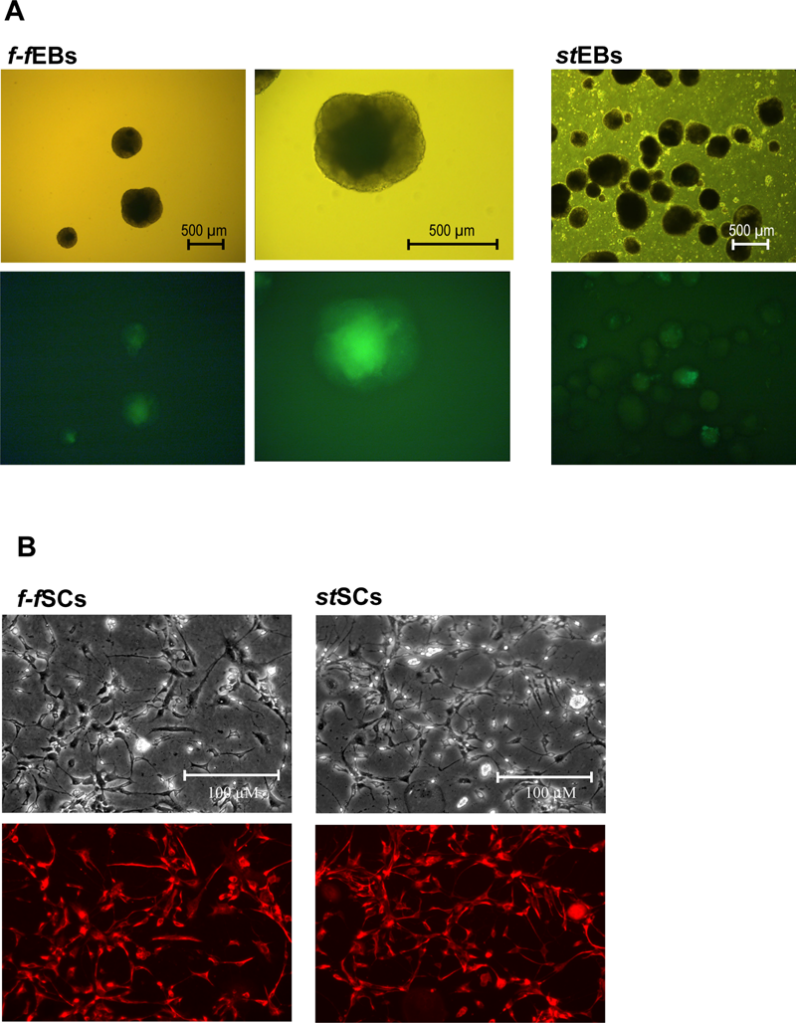
Protein expression analysis of neuronal or cardiomyocyte differentiation markers. A. GFP expression in *f-f*EBs and *st*EBS at day 14 (GFP under the control of αMHC-promoter). B. Immunocytochemical analysis of nestin expression at phase V in *f-f*SCs and *st*SCs.

As an additional control we have utilized murine embryonic stem cells that express green fluorescent protein (EGFP) under the control of α-MHC-promoter. The system allows real time monitoring of ES cell differentiation and green cells could be detected only in cardiomyocytes. The ES colonies were aspirated from feeder cell layers and proliferated as *f-f*EBs in V-96 wells. Substantial green fluorescent protein expression was observed at day 14 following EB formation, indicating cardiomyocyte differentiation in *f-f*EBs and *st*EBS. However, EGFP-expression was observed in every embryoid body developed from aspirated stem cell colony fractions (>100 *f-f*EBs analysed). In EBs cultured under standard conditions only 34 out of 100 *st*EBS exhibited EGFP-expression (Figure 7A).

The time dependent expression pattern of early and late neural/neuronal differentiation markers was comparable in *f-f*EBs and *st*EBs, indicating corresponding differentiation potentials of *f-f*EBs and *st*EBs (Figure 5A). Expression of *fgf5* an early neuroectodermal marker peaked at day 4 during stage III (selection of neural precursors) indicating the onset of neural differentiation at this time point. Expression of this marker was comparable in *f-f*SCs and *st*SCs at all time points, indicating that induction of neural differentiation is not affected by the dissociation of feeder and stem cells.

*Nestin* expression was also comparable in *f-f*SCs and *st*SCs. Although *nestin* RNA expression diminished in both approaches during neuronal differentiation, *nestin* protein expression was verified by immunocytochemistry in differentiated *f-f*SCs and *st*SCs cells at stage V (Figure 5A, B; 7B). Expression of *neurofilament* mRNA was corresponding in *f-f*SCs and *st*SCs and peaked during the late phase of neural precursor selection and was not expressed during neural precursor proliferation and neuronal differentiation. Early expression of the 68 kDa *neurofilament* subunit is in accordance with studies showing a sequential expression of neurofilament subunits in developing neurons [21]. mRNA expression of the late neuronal marker neuron-specific enolase (*NSE)* was distinctly expressed at a late neuronal differentiation stage (Stage V) in both *f-f*SCs and *st*SCs.

**Figure 8:**
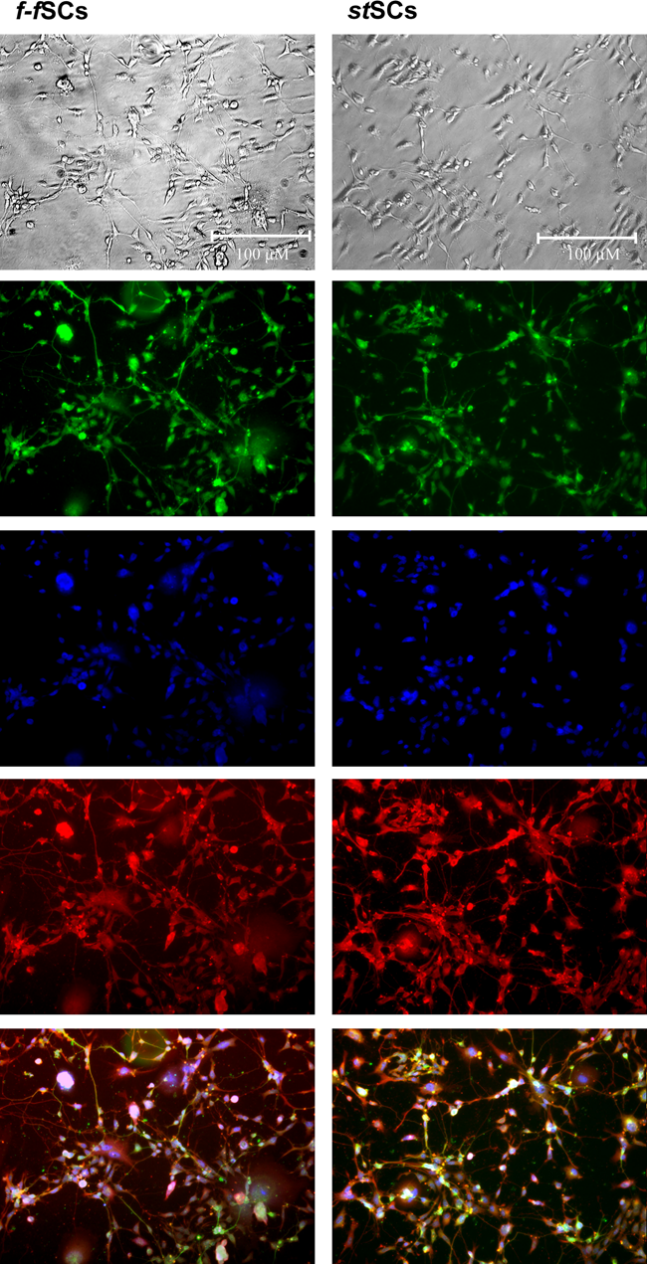
Protein expression analysis of neuronal differentiation markers. Immunocytochemical analysis of microtubulin associated protein 2 (*MAP2;* cyctoplasma, green), neuron-specific protein (*NeuN;* nucleus, blue), and neuron-specific enolase (*NSE*; cytoplasam, red) at phase V in *f-f*SCs and *st*SCs.

This is in accordance with immunocytochemical analysis of neuronal differentiation at stage V. At this stage both *f-f*SCs and *st*SCs developed neurites and/or axonal outgrowth (Figure 7A; B). Differentiated cells expressed neuron-specific protein (*NeuN*; nucleus, blue), microtubulin associated protein 2 (*MAP2*; cyctoplasma, green) and *NSE* (cytoplasma, red). There was no difference in number of differentiated cells or intensity of neuronal marker expression between *f-f*SCs and *st*SCs (Figure 8).

## Discussion

Environmental parameters such as culture conditions can have a distinct effect on the phenotype of stem cells. Cell density [22], culture medium [23] and the choice of feeder cells [4], [24] contribute to stem cell fate.

Feeder cells secrete growth factors required for embryonic stem cell survival and/or proliferation as well as the blocking of spontaneous differentiation to achieve self-renewal [13], [25]. In addition to soluble factors the niche itself might be critical in directing self-renewal and growth of ESCs [15], [26], [27]. In a variety of studies with adult and embryonic stem cells the relevance of cell-cell interactions for maintaining “stemness” has been demonstrated [16], [28], [29]. Hence by substituting feeder cells for conditioned medium or selected growth factors an important if yet not thoroughly analysed aspect of stem cell survival and/or growth is simply abandoned.

The standardized and automated aspiration of feeder-freed murine embryonic stem cell colony fractions established by us permits the initial proliferation of stem cells under optimal conditions that is by co-culturing embryonic stem cells on a supportive feeder cell layer. At the same time feeder cell contaminations that might interfere with later stages of stem cell biology as well as analysis and/or applications can be forestalled.

Our results show that the standardized aspiration of murine embryonic stem cells using an automated system results in embryonic stem cell colony fractions that are completely freed of feeder cell contaminations. Individual feeder-freed stem cell colony fractions were seeded in wells of untreated V-96 plates. Using this method each aspirated stem cell fraction developed into an embryoid body in these wells. However, initially in some wells multiple EBs rather than one discreet EB were detected. The formation of multiple EBs was virtually abolished when the aspiration tool was programmed to release *f-f*SCs closer to the bottom of the V-96 wells.

In a recent publication only forced aggregation by centrifugation induced embryoid body formation of human stem cells seeded in V-96 wells [18]. In this study defined cell numbers were seeded after cells were harvested using collagenase and scraping. While forced aggregation is necessary due to the seeding of disassociated human embryonic stem cells, automated aspiration of stem cell colony fractions does not require cell releasing enzymes, such as trypsin or collagenase. Cells are harvested as aggregated cell clusters by this method. Seeding of intrinsically aggregated cells probably supersedes forced aggregation by centrifugation.

Cross sectional dimensions of feeder-freed embryoid bodies cultured in V-96 wells significantly exceeded the size of embryoid bodies cultured under standard conditions in hanging drops or in V-96 wells during prolonged culturing. This might indicate an inhibitory effect of feeder cells on embryoid body growth. The increased growth of embryoid bodies after day 4 was paralleled by a significantly increased expression of oct4 underlining the augmented proliferative state of *f-f*EBs. In addition to oct4, gata-6 expression (endodermal differentiation marker) was also observed to be enhanced in late stage *f-f*EBs. Enhanced gata-6 expression was concomitant with enhanced expression of cardiomyocyte markers such as gata-4, cTnC and α-MHC in *f-f*EBs indicating a more pronounced initiation of cardiomyocyte differentiation in EBs freed of feeder cell contaminations. Feeder cell contamination is relatively high during embryoid body formation (>7×10^3^ feeder cells/10^5^ stem cells). We therefore assume a modulating effect of feeder cells on EB proliferation or induction of differentiation to be most pronounced during this stage. To what extent the proximate course of stem cell fate might be conditioned by marked feeder cell contaminations during early EB development remains to be elucidated.

Maintenance of pluripotency of stem cell colony fractions cultivated as *f-f*EBs was demonstrated by neuronal differentiation of these cells. Following seeding of EBs in treated culture plates we observed no significant differences in temporal expression patterns and/or intensity of neural and/or neuronal markers during early and late neuronal differentiation of *f-f*SCs or *st*SCs, indicating that separation of feeder and stem cells following stem cell proliferation and growth does not affect the potential of feeder-freed stem cells to differentiate into a variety of tissue specific cells.

The amelioration of an automated cell selection system into a system that allows the aspiration of distinct cell colonies or fraction of colonies from co-cultures by predefined parameters will notably facilitate regenerative and tissue engineering approaches. The complete separation of feeder and stem cells following the proliferation of stem cells on a feeder cell layer will also allow a more detailed analysis of the effect of feeder cells on stem cell differentiation and facilitate the identification of optimal human [3], [30], [31] or genetically modified feeder cell lines [32], [33], [34] for more demanding human embryonic or adult stem cells. Taken into account the significant variability between independently derived human embryonic and adult stem cell lines [35], [36] [18], automated separation of stem and feeder cells will ensure clinical applications of pure stem cells that can be derived from a wider variety of stem cell lines. It also allows for the selection of specific colonies, i.e. after genetic modification of stem cells, and of distinct colony regions if necessary.
